# Remotely-supervised transcranial direct current stimulation (tDCS) for clinical trials: guidelines for technology and protocols

**DOI:** 10.3389/fnsys.2015.00026

**Published:** 2015-03-17

**Authors:** Leigh E. Charvet, Margaret Kasschau, Abhishek Datta, Helena Knotkova, Michael C. Stevens, Angelo Alonzo, Colleen Loo, Kevin R. Krull, Marom Bikson

**Affiliations:** ^1^Department of Neurology, Stony Brook MedicineStony Brook, NY, USA; ^2^Soterix Medical Inc.NY, USA; ^3^MJHS Institute for Innovation in Palliative CareNY, USA; ^4^Olin Neuropsychiatry Research Center, Yale University School of MedicineNew Haven, CT, USA; ^5^School of Psychiatry, University of New South Wales, Black Dog InstituteRandwick, Australia; ^6^Department of Epidemiology and Cancer Control, St. Jude Children’s Research HospitalMemphis, Tennessee, USA; ^7^Department of Biomedical Engineering, The City College of New York of CUNYNY, USA

**Keywords:** tDCS, clinical trials, attention deficit hyperactivity disorder, depression, multiple sclerosis, palliative care

## Abstract

The effect of transcranial direct current stimulation (tDCS) is cumulative. Treatment protocols typically require multiple consecutive sessions spanning weeks or months. However, traveling to clinic for a tDCS session can present an obstacle to subjects and their caregivers. With modified devices and headgear, tDCS treatment can be administered remotely under clinical supervision, potentially enhancing recruitment, throughput, and convenience. Here we propose standards and protocols for clinical trials utilizing remotely-supervised tDCS with the goal of providing safe, reproducible and well-tolerated stimulation therapy outside of the clinic. The recommendations include: (1) training of staff in tDCS treatment and supervision; (2) assessment of the user’s capability to participate in tDCS remotely; (3) ongoing training procedures and materials including assessments of the user and/or caregiver; (4) simple and fail-safe electrode preparation techniques and tDCS headgear; (5) strict dose control for each session; (6) ongoing monitoring to quantify compliance (device preparation, electrode saturation/placement, stimulation protocol), with corresponding corrective steps as required; (7) monitoring for treatment-emergent adverse effects; (8) guidelines for discontinuation of a session and/or study participation including emergency failsafe procedures tailored to the treatment population’s level of need. These guidelines are intended to provide a minimal level of methodological rigor for clinical trials seeking to apply tDCS outside a specialized treatment center. We outline indication-specific applications (Attention Deficit Hyperactivity Disorder, Depression, Multiple Sclerosis, Palliative Care) following these recommendations that support a standardized framework for evaluating the tolerability and reproducibility of remote-supervised tDCS that, once established, will allow for translation of tDCS clinical trials to a greater size and range of patient populations.

## Introduction

Transcranial direct current stimulation (tDCS) is a noninvasive brain stimulation (NIBS) technique that utilizes low amplitude direct currents (typically less than 2.5 mA current and 0.08 mA/cm^2^ average electrode current density) to induce changes in cortical excitability. Advantages of tDCS compared to other methods of NIBS, such as transcranial magnetic stimulation (TMS), include ease-of-use, low cost, and tolerability (Vanneste et al., 2010). Using clinical grade equipment and following strict protocols with trained operators, tDCS has been tested in hundreds of clinical trials and is considered to be both safe and well-tolerated for study in a wide range of subjects (Nitsche et al., 2008; Brunoni et al., 2012b; Kalu et al., 2012).

tDCS can influence sensory, motor, cognitive and psychiatric processes that could be applied directly to the treatment of common yet refractory symptoms that represent major areas of unmet treatment need, such as depressed mood, pain, fatigue, sensory and motor recovery, and cognitive impairment (Ball et al., 2002; Fregni et al., 2006; Mori et al., 2010, 2013; Andrews et al., 2011; Acler et al., 2013; Brunoni et al., 2013b; Cuypers et al., 2013; Bennabi et al., 2014), typically occurring in the context of a neurologic or psychiatric condition.

### Remote Delivery Will Expand tDCS Clinical Study

Neurophysiologic and clinical trials with tDCS increasingly reinforce that efficacy increases with multiple sessions (Mori et al., 2010, 2013; Acler et al., 2013; Ferrucci et al., 2014; Meesen et al., 2014; Tecchio et al., 2014), with effects thought to be cumulative. Many clinical investigators apply tDCS in conjunction with a behaviorally-based treatment approach to improve outcome (Demirtas-Tatlidede et al., 2013; Martin et al., 2013b; Brunoni and Vanderhasselt, 2014; Brunoni et al., 2014; Flöel, 2014), which must be repeated alongside tDCS. The clinical utility of tDCS must be established through trials that are sufficiently-powered, with adequate dose (Peterchev et al., 2012) and session number, and with precise protocol control. However, multiple sessions require subjects to repeatedly travel to the clinic for each treatment, placing significant and often insurmountable burden to patients and their caregivers, at the same time associated with significant provider time and cost, especially as the sample size increases (Brunoni et al., 2012a; Holland and Crinion, 2012; Ferrucci et al., 2014; Meesen et al., 2014; Shiozawa et al., 2014; Vaseghi et al., 2014). For example, in a sample of 64 subjects treated for depression, Loo et al. administered 30 sessions across 6 weeks (Loo et al., 2012), followed by up to 20 maintenance treatment sessions spaced over 6 months (Martin et al., 2013a). Similarly, during the 6 month follow up of a depression trial, Valiengo and colleagues showed a dropout rate of 17 of 42 subjects—with almost all dropouts citing the burden of regular visits to the clinic (Valiengo et al., 2013).

A remedy to this feasibility problem is controlled remote tDCS application, with a common protocol that ensures safe, well-tolerated and reproducible remotely-supervised tDCS. Combining this remote approach with behaviorally-based treatments (e.g., cognitive or physical rehabilitation exercises completed at home, or behavioral therapy delivered through remote, web-based platforms) will allow investigators to reach subjects either through satellite clinic locations or directly from their home or care facility.

While there has not yet been a clinical trial involving remotely-supervised tDCS, the home use of tDCS over a 3 year period has been reported as both safe and effective in the case of a patient with schizophrenia through 20 minute daily sessions administered by a medically licensed caregiver (Andrade, 2013).We propose here a protocol for remotely-supervised delivery of tDCS for clinical trials, with the expectation of maintaining the same level of uniformity and compliance that would be seen with tDCS sessions administered in the clinic. This step represents an extension of currently-accepted tDCS methodology to allow for trials to include more subjects and to remove any logistical limits on the number of sessions studied.

The approach proposed for remotely-supervised tDCS can be applied to double-blind trials. tDCS clinical trial devices have been developed which deliver active or sham stimulation depending on the individual subject code keyed in, leaving both device operator and subject blinded to treatment assignment. There is no reason why blinding should be any less available when the subject (or proxy) operates the device under remote supervision.

### The Potential and Limitations of tDCS Away From Clinic

Below, we outline a set of what we consider to be essential features for this next step of remotely-supervised tDCS delivery. While the tDCS may be self- or proxy-administered, an important distinction is the difference with this controlled and remotely-supervised extension as compared to direct home use. One recent report described the challenges of prescribing tDCS directly for self-administered home use, without remote supervision. Using a crossover design to treat pain in patients with trigeminal neuralgia, investigators (Hagenacker et al., 2014), instructed subjects and one other adult to apply tDCS at home using a device pre-programmed to alternate active and sham stimulation across 14 sessions over 2 weeks, recording any adverse effects in a diary. All subjects tolerated the stimulation well with no adverse events reported; active treatment was found to be effective in reducing pain. However, many subjects reported difficulty with the tDCS application with an associated high dropout rate (41%). The authors concluded that a more specific and detailed education and training protocol could improve delivery. Of further concern, there was minimal guidance or structure during the course of sessions, possibly leading to variability of method across subjects and no explicit safety monitoring.

It is clear that a structured protocol is needed to identify subjects who are appropriate candidates for remote study, and to ensure that they—or a proxy who will administer the tDCS for them (e.g., caregiver)—are adequately trained. This training and certification, must be gauged against the usability of the (specially-designed) device and headgear, the likelihood that the subject may fluctuate in ability to operate the equipment (e.g., due to fluctuations in physical or mental state), the risks associated with the specific trial, and other relevant study factors such as the nature of ongoing monitoring.

As a qualifier, specialized equipment designed for this remote study purpose is a minimum requirement. State-of-the-art clinical tDCS equipment and accessories have been developed and validated in controlled clinical trials, with trained operators. It is not safe or prudent to simply provide subjects with specialized clinical equipment, including devices and headgear, not designed for home use. Doing so without accounting for variability in skill, training, and environment puts subjects at risk and compromises reproducibility (e.g., dose control). The distinction between medical equipment designed for professional operators vs. subjects and their caregivers is evident across physical medicine, and is no less important for tDCS. It is misguided to conflate the inherent simplicity and tolerability of clinical tDCS with the assumption that devices not designed for subject use applied by untrained operators pose no risk (Bikson et al., 2013) and allow for reproducible protocols (Peterchev et al., 2012).

Our guidelines are built around ongoing supervision in real-time, even with subjects who have completed training and demonstrated competency to self-administer. While the level of supervision may vary across patient group or risk level at the subject level, we believe that consistency is essential for every tDCS session to ensure safety and tolerability, as well as to maintain the standards of administration set by tDCS sessions in the clinic.

## Requirements for Remotely-Supervised tDCS: Essential Features

*The recommendations listed here are governed by the principle that remote tDCS administration must be safe, structured and reproducible across study sites. Items are generally categorized by training of staff and subjects/caregivers, design of headgear and stimulators, and ongoing monitoring. These items are interrelated and are itemized here for emphasis of key points—in clinical trial design they will be implemented holistically*.

### Training of the tDCS Research Staff

**Figure 1:**
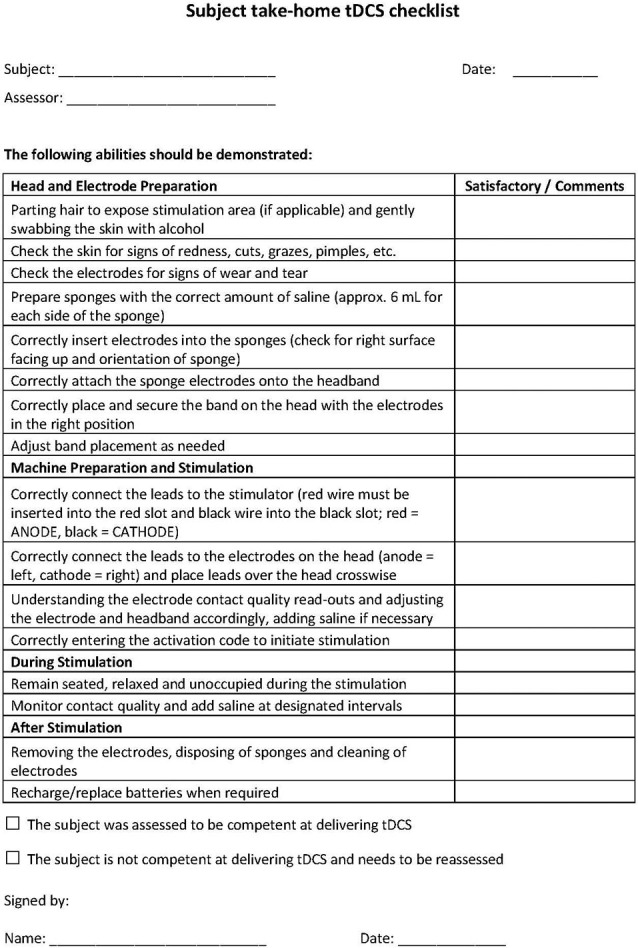
**Example of subject checklist**.

Study research staff will first be trained in tDCS treatment technique and in administering tDCS to others. Staff will be trained using standard-operating-procedures (SOP) specifying all subject interactions as well as device usage. In terms of subject interaction, study research staff will be trained to monitor for study-specific “stop” criteria following a decision-tree-based flowchart that allows for evaluation of eligibility to continue at each progressive step. At baseline visits (in clinic) tDCS research staff will complete a checklist to screen subjects for at home tDCS (see Figure 1 for an example).

Following training on screening procedures, study research staff will be briefed on the usage of the device and the location of headset placement. In addition, selected research staff will be trained with and have access to a guide that details (e.g., the device manual), in a step-by-step fashion, the procedures that are required to configure and program devices prior to release to subjects, for example programming device unlock codes for one-time use to be provided prior to each session. Prior to releasing a device to a subject, selected research staff must program the intensity, duration, and condition (active or sham) of each planned session, along with code that limits the number and frequency of sessions. In the case of code-based session release, research staff will be instructed to withhold any one-time use codes until subject device placement and setup are deemed appropriate.

**Figure 2:**
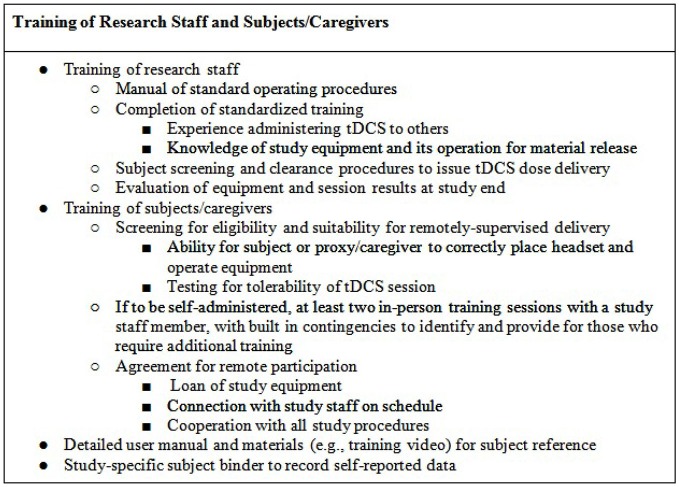
**Guidelines for training**.

Research staff will be trained in the preparation and testing of any devices and accessories (e.g., kits) release to subjects, as documentation of material release. Through the duration of the study, technicians will be trained to monitor subjects for any unexpected adverse events (e.g., atypical discomfort) or misuse of the device. At the end of the study, technicians will be instructed on how to evaluate retrieved physical device materials (e.g., headgear/stimulator condition, expendables) and download any data stored on the device (e.g., completion codes for each subject’s study sessions, stimulation history of each code issued) as relevant to confirm compliance (Figure 2).

In addition to training specific to the implementation of remotely-supervised tDCS as describe above, the clinical research team needs to be skilled in other aspects of clinical trial methodology, including screening of subjects, monitoring of subject progress and evaluation of outcomes.

### Initial and Ongoing Assessment of the User’s Capability to Participate in tDCS Remotely

**Figure 3:**
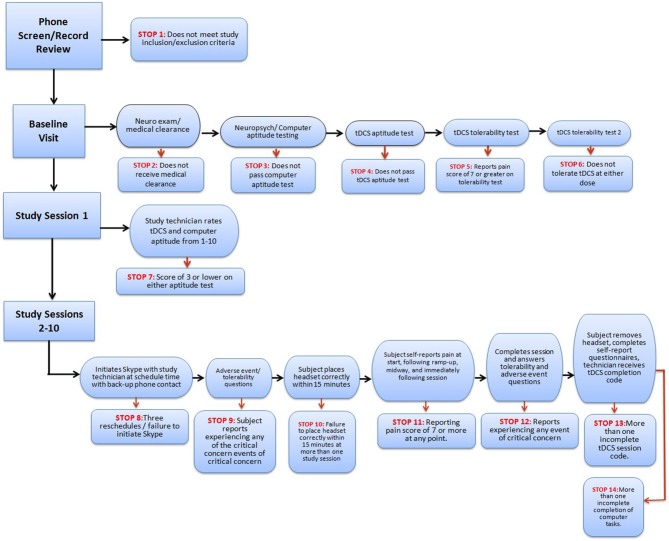
**Example of study flowchart with stop criteria**.

A precisely defined protocol (decision-tree-based flowchart) will be closely followed through the duration of the study for each subject. Study “stop” criteria (Figure 3) will be reviewed at each stage of the trial: screening, baseline, study sessions, and follow-up. If stop criteria are met at any time throughout the study, the session and/or trial participation will be terminated as specified in the protocol. Following an initial screening, at least one baseline visit will confirm the subject’s tDCS aptitude and tolerability. Before any subject is sent home with a device, it will be determined whether the subject-or designated proxy- can properly follow the steps to ensure correct electrode preparation and placement, low impedance and safe removal of the device, and whether the subject can tolerate the stimulation period.

Throughout the study, general compliance will be maintained through stop criteria that exclude subjects who continually fail to set up a device properly or fail to attend regularly scheduled sessions. In addition, if any subject reports an unexpected level of discomfort, pain, or desire to withdraw, the session and/or ongoing study participation may be terminated. At the end of the study, the record of completed and uncompleted sessions, recorded by the device itself, will be reviewed for all subjects who successfully met study criteria. This will be used to determine both individual subject and overall trial compliance based on pre-determined criteria.

### Supportive Training Procedures and Materials (Including Assessments of the User and/or Caregiver)

The protocol will be supported by a manual of operating procedures for study staff. For designs with remote supervision of self- or proxy-administered tDCS, that there be at least two in-person training sessions with study staff, including training and practice for the real-time remote supervision procedures (e.g., a training session with a study staff member that includes connection to another remotely-located study staff member via secure internet-based video conference).

For subjects who meet criteria for study continuation, subsequent sessions will be completed with the remote technician alone, with the nature and length of ongoing monitoring to be specific to the subject or trial. For instance, some subjects may require remote monitoring indefinitely, while other trials may use remote monitoring for a training period only.

The protocol will include sequential safety, tolerability and compliance “stops” administered by the supervising study staff throughout the course of the treatment sessions to ensure appropriate use. For instance, potential concerns include failure to place the headset and electrodes correctly, selecting incorrect electrode polarity, inadequate electrode saturation, lack of compliance, and loss of precision in electrode placement.

Each subject will be provided with supportive and readily-accessible training materials (Figure 2). These should include an instruction manual (potentially supported with an instructional video) and a method to self-report (electronic or binder). These materials are designed as a resource to the subject to reference or confirm study details. In addition, they serve the purpose of remotely training subjects or caregivers who look to clarify items of set-up or device usage. The self-report binder will provide the subject with forms that measure adverse events, assess tolerance, along with study-specific measures (e.g., mood). While the manual and video (if used) will serve as ongoing training procedures, the self-report forms will enable the subject and caregiver to assess the experience throughout the study. Any remotely-delivered adjunctive behavioral therapy (e.g., cognitive or physical training) will be trial-specific with a supplemental protocol and procedures.

### Simple and Fail-Safe Electrode Preparation and Positioning

The essential aspects of reproducible tDCS dose (defined in Peterchev et al., 2012) are electrode preparation and montage (addressed in this section) and waveform (addressed in the next). Remotely-supervised tDCS administration would reduce barriers to reliably apply tDCS away from the treatment center.

**Figure 4:**
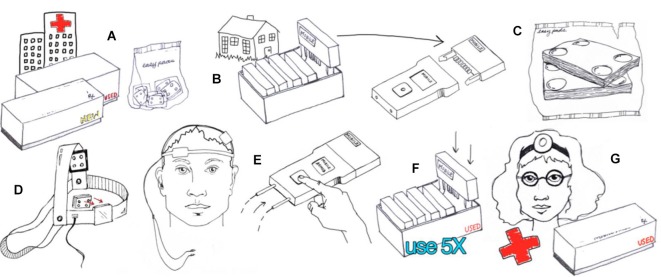
**Example of hardware-based waveform control**. Users are provided with an individual device and accessories such as the 5x-Session Home Kit **(A)**The subject checks in with the supervisor before and after each session **(B)**. The supervisor unlocks operation before each session by providing a code **(B)**. The subject enters only the code provided with no access to device programming or stimulation settings. The subject uses custom fit headgear to position electrodes **(C,D)**. The device automatically collects compliance data and may also prompt the user for information **(E)**. Details of implementation will be customized to each clinical trial while maintaining the principles of supervised neuromodulation **(F, G)**. *(Image courtesy of Soterix Medical Inc.)*

**Figure 5:**
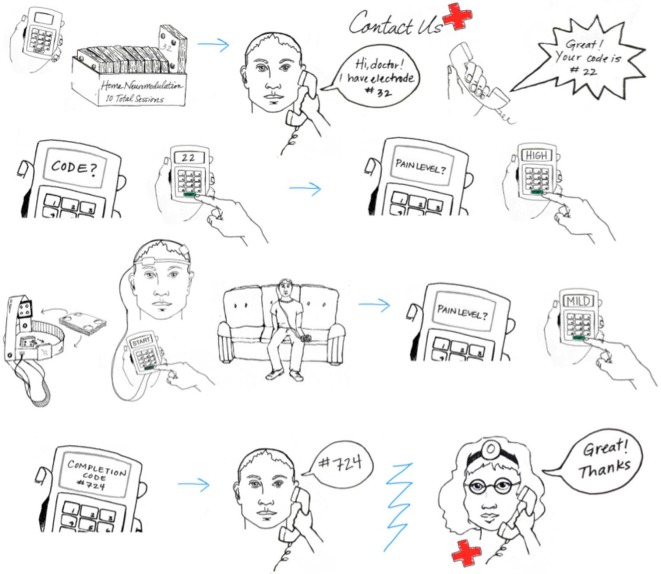
**Example of software-based waveform control**. Users are provided with an individual stimulator, a discharge key, and accessories such as the 5x-Session Home Kit. The supervisor limits stimulation by programming the discharge key. The subject plugs the discharge key into the device and presses a single button to activate stimulation. The subject uses custom fit headgear to position electrodes. The device automatically collects compliance data which is stored in the discharge key. Details of device implementation will be customized to each clinical trial while maintaining the principles of supervised neuromodulation. *(Image courtesy of Soterix Medical Inc.)*

The headgear should be designed to allow simple and consistent placement of electrode at desired locations on the scalp (Figures 4, 5)—for example headband snaps for the sponges to be placed on the headband to ensure consistent correct placement.

Subjects will be screened for aptitude with the specific headgear as part of inclusion/enrollment. Participation is contingent on the ability to properly apply the headset and correctly operate the device. The headgear may be marked in a way that facilitates reliable setup, for example the headset will be labeled “RED” and “BLACK” to confirm that color coded cables are properly placed, ensuring anode (RED) and cathode (BLACK) placement (white can be substituted for red if color blindness is a concern). The headset should be designed to fit reliably on the head, for example designed with a market to align with the bridge of the nose at midline. Headsets may come in multiple sizes and/or allow adjustment by research staff. The device includes an impedance meter and will not allow access until headgear and electrodes are accurately and correctly placed.

**Figure 6:**
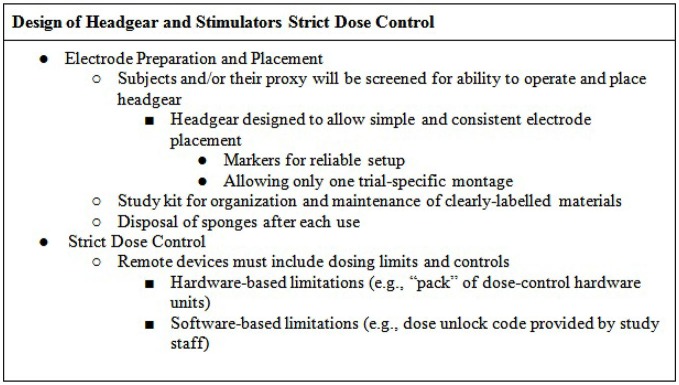
**Guidelines for study equipment**.

The headset should be designed to minimize error in electrode placement, ideally allowing just the montage designed for the specific trial (Figure 6), e.g., bifrontal montage (for dorsolateral prefrontal cortex target application) or M1-SO montage (for motor cortex target applications).

Subjects/proxies should be provided with kits which include all of the necessary supplies for the entire study or until expendable can be replenished (by either a visit to the clinic or visit by study personnel to the subjects’ home or care-facility). Components should be labeled properly in order to simplify application, minimize confusion, and prevent error (e.g., mark each pair of sponges by session number). The subject manual should clearly explain the contents of the kit and the correct use of each component.

Also of note, re-use of sponges across subjects is not hygienic. Re-use of the sponge across sessions, even when cleaned, is not recommended for supervised home use- single-use sponges increase tolerability, cleanliness, and reproducibility. Medical grade sponges are required to comply with biocompatibility requirements, and labeling for re-use would require testing under the intended conditions of use—which are complex and variable in the case of home-application. Reliable control of cleanliness, much less contamination, would prove difficult in home use (would not for example be reliably detected by impedance check or remote observation).In addition, attempt to remediate these sponge re-use concerns would increase the operator and patient burden for monitoring and preparation (e.g., concerns about storage, residue from skin/hair product).

### Strict Dose Control for Each Session

Control of stimulation parameters is the second essential aspect after electrode placement. For tDCS essential parameters are the intensity, duration (along with any ramp), and condition (active or sham) of stimulation. For multiple sessions, the number and interval of sessions is critical. For remotely-supervised tDCS, limitations on the number and timing of sessions, along with control of each session intensity and duration is pivotal (Figure 6). Providing subjects with clinical stimulators that do not limit either is not safe or supportive of reproducible protocols.

Two general approaches to control stimulation parameters under remotely supervised-tDCS can be considered. In the first case, “hardware” based limitation (Figure 4) provides subjects with equipment that is pre-programmed to provide a limited number of sessions with limited interval. For example, the stimulator will provide only 10 sessions, with pre-set intensity, duration, and condition, with a maximum of one session every 23 h. Once 10 sessions have exhausted, the device no longer provides output until the entire hardware or a component (storage disk) is replaced. With “hardware” limitations, it is not necessarily possible to remotely stop use before the 10 sessions are activated. The Soterix Medical/Neuroconn Mobile transcranial Direct Current Stimulator (Mobile) and Magstim/Neuronica HDC-Kit are example of “hardware” based limitation. Subjects or caregivers are required to return to the clinic to replenish expended hardware—supervisors may be provide a “pack” containing multiple dose-control hardware units (e.g., 10 storage disks with 10 sessions each for 100 sessions) but this effectively extends the window where control of dose is limited.

In the second case, “software” based limitation (Figure 5) subjects are provided with a device which is deactivated until a code is provided by the research staff. The code typically unlocks a single session which a pre-set duration and intensity. After a single discharge, the device is inert until a new code is provided. This allows the remote supervisor to tightly control compliance, for example not providing a code until proper electrode set-up is confirmed by video, provides an opportunity for supervisor to directly interact with subject and gather feedback and data for each trial. In this situation, the remote supervisor may vary the provided code based on subject experience. Soterix Medical transcranial Direct Current Stimulator mini-Clinical Trials system (Mini-CT) is one example of “software” based limitation. Given stimulators can be programmed with excess of codes (e.g., thousands), there is no need for subjects to return to the clinic to replenish hardware.

In both “hardware” and “software” based governing for remote supervision, the subject or caregiver does not program session intensity of duration which enhances safety and reproducibility. In case of blinded treatment, the subject may not know which device/code is real or sham treatment. Double blinding may also be implemented by blinding the remote supervisor.

The user manual should clearly explain the hardware contents of the kit and the correct set-up of the device, such as entering a code or reading the display.

### Ongoing Monitoring for Compliance

**Figure 7:**
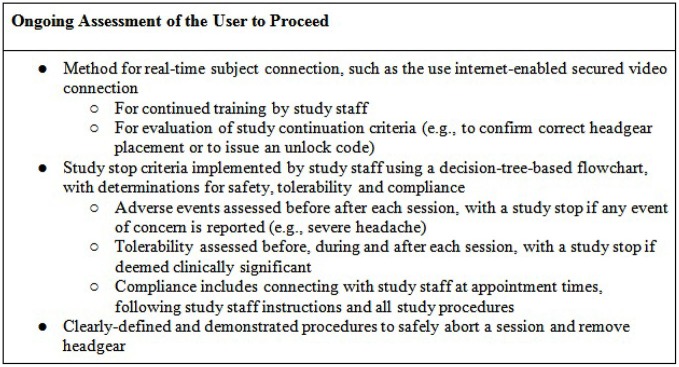
**Guidelines for ongoing assessment**.

While in-person monitoring will be completed during the initial sessions, subjects may be monitored and/or contacted to confirm compliance throughout the remaining sessions. Subject interaction will be maintained (e.g., through secure video) to quantify compliance (device preparation, electrode saturation/placement, stimulation protocol) with corresponding corrective steps. As an example, secure video will be scheduled and maintained with each subject daily. Visual confirmation will ensure proper set up, appropriate home environment, and acceptable impedance. Following these checks during monitoring, subjects will be given their one time use code for that session (Figure 7).

### Ongoing Monitoring for Treatment-Emergent Adverse Effects

Before starting a session, study staff will ask the subject about any adverse events since the last session, with clearly defined stop criteria if anything is reported that is of clinical concern related to safety or tolerability. In addition, subjects will be asked to report pain and/or discomfort level at set points during the stimulation. If pain crosses a given threshold, there will be an immediate stop in place in order to prevent any risk of skin burn (Loo et al., 2011) and the supervising staff will assess whether this has occurred due to suboptimal treatment technique. If tDCS causes pain above the given threshold despite adequate treatment technique, subjects will be withdrawn from the study. After the session, subjects will be again assessed by study staff for any adverse events. Subjects will also record their experience in the study-specific materials.

### Guidance to Identify and Implement Discontinuation of a Session and/or Study Participation (Including Emergency Failsafe Procedures) Tailored to the Treatment Population’s Level of Need

Finally, during training and in reference materials, subjects will be instructed to abort the session if they need to immediately discontinue stimulation by allowing ramp down and then removing their headset. These instructions will be used if any subject reports significant discomfort or other adverse event, otherwise needs to discontinue a session, or if study staff determines that the session should be discontinued.

## Examples of Clinical Trial Applications and Population-specific Adaptations

*The authors’ interest in remote (non-clinic) tDCS for clinical trials has facilitated effort to develop study protocols for specific patient populations. Below, we provide examples of population-specific considerations that may navigate tailoring the guidelines described above to specific populations with differing limitations and needs. These summaries are not intended to be exhaustive but rather illustrate salient features of applying the above eight principles to specific applications. Once population specific protocols have been implemented, and the feasibility of these methods has been confirmed within the target groups, more expansive clinical trials can apply them to study the effectiveness of the treatment*.

### Attention Deficit Hyperactivity Disorder (ADHD)

The immediate facilitatory effects of tDCS on cognitive abilities often seen impaired in ADHD has prompted interest in whether or not the technique might have a therapeutic effect. For instance, a recent study of ADHD-diagnosed children in another experiment showed that 0.75 Hz oscillating tDCS increased EEG-recorded slow wave oscillation during sleep, and subsequent memory recall the next day (Prehn-Kristensen et al., 2014). One brief tDCS trial in non-ADHD subjects showed better response inhibition task performance after 5 consecutive days of training with concurrent 1.5 mA anodal tDCS stimulation than training alone (Ditye et al., 2012). The latter suggests that tDCS paired with cognitive training/rehabilitation techniques might have the highest potential for efficacy in minimizing ADHD-related behavioral dysfunction. However, previous trials that combine these therapeutic approaches typically last 1–3 weeks, making clinic visits impractical for a typical family that contends with school demands and extra-curricular activities, worsened when there is more than one school-aged child in the home. Any clinic-based tDCS trial for ADHD not only would be plagued by poor compliance and high dropout, but it is also likely that only the most motivated of families and subjects would complete treatment, complicating generalizability and efficacy inferences.

Remotely-supervised tDCS performed is an obvious approach to circumvent the main obstacles, but the age and behavioral characteristics of the patient population introduce unique issues to any home-based tDCS clinical trial. ADHD is a behavioral disorder whose hallmarks are distractibility, restlessness, and motivational issues–all of which must be managed effectively within a standardized tDCS delivery protocol. Although self-administered tDCS for adults diagnosed with ADHD certainly is feasible, it is more likely that trials will consider tDCS with older children or adolescents, as tDCS has been shown to be safe with these age groups. However, most institutional review boards will not likely approve trial protocols where youth are asked to set up and administer tDCS themselves, suggesting effective trials must plan to overcome and troubleshoot issues arising from parent training to administer tDCS properly. Such issues include effective parent screening/training, attention to how dyadic interactions influence motivation, and special care in assessing compliance and outcome. To accommodate these special considerations, we recommend a trial design that at minimum includes: (1) a clinic visit for consent, clinical assessment, and training with particular attention paid to educating families that treatment must be a “whole family” cooperative effort, (2) an “at home” visit prior to treatment so that research staff can assess and advise tDCS equipment set up and other technical issues, (3) careful consideration of whether every tDCS session should be remotely-supervised (e.g., via video teleconferencing) to ensure optimal protocol adherence; and (4) formal consideration in trial design of how ADHD-related behavior interacts with both treatment delivery, motivation, or outcome.

Some specific recommendations for ADHD protocols include:

-* Parent preparedness, training, and motivational issues*: Informed consent for a remotely-supervised tDCS trial should emphasize the multiple types of training required for parents who will administer tDCS. At minimum, parental tDCS technical proficiency should be demonstrated as suggested above, with at least 1 clinic visit and 1 remotely-supervised session, if not at each daily session. If computer-administered cognitive training techniques will accompany tDCS stimulation, separate training should be provided in how to start, stop, and evaluate the successful implementation of such techniques. In addition, parent training should be provided in how to properly motivate their ADHD-diagnosed child. Obviously, an important goal is to avoid punitiveness or coercion that might occur if parents are more motivated than their children, but it is equally as important is to standardize and measure motivation across the trial. For example, simple behavioral contingency management techniques should be considered as standard, especially for younger age groups. Protocol-specific guidelines on how and when to provide positive reinforcements should be made explicit; their use should be quantified by trial staff weekly.
-* Dyadic interaction impact*: It is not yet known what interaction styles or personality factors might make some parents more effective in operating and supervising tDCS at home than others. Until such study has been done, we recommend that each trial should make effort to formally assess the nature of parent-child dyadic interactions or familial relationships styles to see if they moderate treatment compliance or outcome.
-* Preparation for tDCS delivery and Monitoring of Potential Treatment Barriers*: Homes with children are different than those without. For tDCS with a child or teenager, tDCS should be set up in a specially-designated quiet room and performed without distraction or interruption, preferably with the cooperation of the whole family during treatment delivery to reduce distractions. Protocols should set clear guidelines for whether or not breaks are permitted during training and set guidelines for them. A standardized compliance checklist should be used for each session, which emphasizes whether ADHD-related behaviors that could have interfered with treatment occurred and how they were managed. Any concurrently-done computer-administered cognitive training ideally will be internet-enabled. This will allow research staff to remotely verify that any exercises were set up and run correctly, as well as offers the potential for any behavioral performance data to be uploaded for secure storage at the clinic site after each treatment session.
-* Careful Choice of Outcome Measures*: Although it is likely that the primary outcome measures of any tDCS trial would be performance on tests of a specific cognitive domain, (either because it is specifically targeted by concurrent cognitive training or merely because it is one of several domains often impaired in ADHD), secondary outcome measures should include ADHD symptom severity checklists or ADHD “problem behavior” inventories (e.g., Brown ADD Scales). Because of the subjective nature of the latter types of assessments, it is important to use multiple respondents (e.g., parent and teacher). Similarly, the most effective trials will include appropriate placebo conditions in order to mitigate expectancy effects.

### Depression

There is a long history of the use of “brain polarization”, essentially an earlier form of tDCS often given at lower stimulus intensities, to treat depression (Arul-Anandam and Loo, 2009). Over the last decade, in the context of the emergence of brain stimulation treatments for depression (e.g., transcranial magnetic stimulation) and developments in tDCS equipment, methodology and scientific understanding, interest has rapidly emerged in tDCS as a treatment for depression. tDCS has been applied both to treatment resistant depression (i.e., in subjects who have failed to improve after treatment with antidepressant medication) and to non pharmacotherapy resistant depression, as some patients may prefer a non medication form of treatment. From 2006, evidence from several placebo-controlled, randomized clinical trials has indicated significant antidepressant effects for tDCS (Loo et al., 2012; Brunoni et al., 2013a; Shiozawa et al., 2014). A key barrier to the widespread use of tDCS for this common disorder is the requirement to travel to a treatment center for multiple sessions as repeated treatment sessions, typically given on consecutive weekdays over several weeks, are often required to optimize clinical response. For example, the Loo et al. (2012) study suggested that extension of the treatment period from three to 6 weeks resulted in greater clinical improvement. After the acute treatment course, ongoing “maintenance” tDCS, given weekly to fortnightly, has also been reported to be useful in maintaining improvement gained in the acute phase and preventing relapse of depression (Martin et al., 2013a).

Several specific issues need to be considered when applying tDCS in depression trials:

- An important precaution in this patient group is suicide risk, which may fluctuate from day to day and should be closely monitored. It is unknown if tDCS may lead to increased suicide risk early in the treatment course (as has been proposed with antidepressant medications), either due to improved motivation, or specific treatment-related effects.
- Dose-control, i.e., control of access and restriction of stimulation to pre-programmed stimulation is important as a safeguard so that the device cannot be used for deliberate self-harm.
- The research team should closely monitor fluctuations in mental state, which may impinge on the subject’s ability to adequately perform the tDCS procedure at home, for example, due to changes in motivation or concentration. Thus, it is possible that a subject initially assessed as capable of performing remotely supervised tDCS at home, later becomes unable to adequately continue treatment at home—contingencies should be made for this event. Conversely, given findings that tDCS improves information processing speed in depressed patients (Loo et al., 2012) it is also possible that subjects initially assessed as not capable of performing remotely supervised tDCS at home, who may need to commence acute treatment at the treatment center, may later be able to continue the acute treatment course and ongoing maintenance treatments at home under supervision.
- As part of the initial assessment of suitability for home-based, remotely supervised tDCS, a thorough psychiatric assessment by an experienced clinician is required, evaluating not only mood, but also personality style, current stressors and social and family support. All of these factors should be considered in evaluating a person’s ability to comply with tDCS procedures, likely fluctuations in mental state and risk.
- The majority of depressed patients presenting for treatment are likely to be on a combination of psychotropic medications, including antidepressants, atypical antipsychotics, anxiolytics, lithium and anticonvulsant mood stabilizers. Further, alterations in concurrent medication treatment may occur frequently common in order to manage side effects, achieve better antidepressant response or provide symptomatic relief (e.g., sleep, anxiety). As there is evidence that anticonvulsant and benzodiazepine medications may affect the efficacy of tDCS, these medications are best avoided if possible. The tDCS team should maintain close liaison with the subject’s treating doctor(s) throughout the tDCS treatment course, to optimize concurrent medications and to establish procedures for clear and prompt communication about any alterations in medications.

### Multiple Sclerosis (MS)

For those living with MS, treatments using tDCS have the potential to directly alleviate common yet refractory symptoms including pain, fatigue, depression, and sensory and motor dysfunction (Palm et al., 2014). An important first step is to establish the procedures for feasible at home use, with consideration of potential motor and cognitive impairments.

For this patient population, the emphasis is on extensive and ongoing screening procedures, as well as streamlined and simplified equipment design and set up. To ensure compliance, safety, and assist with training, a secure, daily video monitoring protocol should be implemented.

Further recommendations for MS protocols include the following:

- Screening is an important first step as remotely-supervised tDCS will not be appropriate for all MS patients. A treating neurologist should ensure that the subject has potential suitability for operating the headgear and device. This would include cutoffs for minimal neurological (motor) and cognitive function. For instance, severe motor impairment can be screened for using the Expanded Disability Status Scale (EDSS) score (Kurtzke, 1983), excluding those with scores of 6.5 or greater. In addition, subjects should be excluded from a remotely-supervised protocol if any visual, auditory, or motor deficits prevent the ability to understand the study instructions or operate the device or laptop computer (Figures 1, 3). Minimal cognitive ability for participation could also be defined, for example by establishing cutoffs using the Brief International Cognitive Assessment in MS (BICAMS; Benedict et al., 2012) to exclude those with severe cognitive deficits.
- The study device should be simple to operate and engineered to unlock single tDCS sessions through a one-time use passcode. Headgear should also be simplified for use. For example, headgear should be modified with a hat-like design to ensure minimal motor requirements. In addition, device kits should be prepared and organized to ensure maximal ease in terms of sponge pocket set-up and daily materials.
- Clinical and research staff should be trained through a study technician manual on the device setup (programming sessions, retrieving session codes), headgear placement, and subject interactions (requirements for a successful session, items that qualify as “stop” criteria, and troubleshooting). In addition, research staff should be trained on how to safely abort a subject session and on procedures to reestablish video connection if lost.
- Subject training should occur during an initial in-person training session where each subject will view an instructional video, practice the technique in full and ultimately complete their first tDCS session. Beyond training, these initial visits will ensure inclusion and exclusion criteria are met. Subjects must have a suitable home environment (e.g., distraction free location and space to complete the sessions) access to internet, and the ability to commit to tDCS sessions.
- An instructional video should support training and implementation, be accessible to the subject throughout the study, and cover all details necessary to complete a session. The video should contain information on general materials, a step by step guide to set up the device, how to abort a session in case of emergency, and the means of properly ending a session and disposing of materials.
- Stop criteria (assessed at baseline, during sessions, and at follow-up) should be used as a gateway for the subject to proceed at each step (Figure 3). The protocol should be designed with a series of checkpoints to be met to address compliance (attendance, ability to complete the procedures as instructed, following study guidelines) and tolerability (at any time if any predefined events are reported or if pain crosses a threshold).

### Palliative Care

Palliative care is an interdisciplinary model of care for patients with serious or life-threatening illnesses. The goal of palliative care is to manage symptoms of the disease and to mitigate illness burden for the patient and family from the time of diagnosis until the end of life. In the U.S., palliative care is available through both hospital-based and community-based palliative care programs (Hauser and Kramer, 2004; Defilippi and Cameron, 2010; Connell et al., 2013; Kamal et al., 2013). An overall goal of community-based palliative care is to provide adequate support in symptom management, and psychological and spiritual support to the patient and family so that the patient can remain at home, even during the terminal phase of the disease. Therefore, the development of novel home-based approaches for symptom control is highly relevant for this patient population.

Although outcomes targeted in existing tDCS studies, such as pain, mood, sleep, cognitive performance, or overall quality of life, are highly relevant for palliative care patients, there are numerous barriers of an access of palliative care patients to the tDCS studies. The burden of repeated visits to the research facility in order to receive the tDCS application has been among the major obstacles. Therefore, the development of the mini-CT tDCS enabling home-application provides an opportunity for the palliative care patients to be included in tDCS studies. However, designing study protocols of remotely-supervised tDCS for palliative care patients requires specific considerations, such as the following:

-* Involvement of family caregiver*: Palliative care subjects frequently rely on an assistance of family caregivers. Therefore, it is likely that the home-based tDCS application will be delivered by the family caregiver rather than self-performed by the subject. Thus, both the subject and the family caregiver may need to be included in the study.
-* Minimizing burden to the subject and the family caregiver*: It has to be kept in mind that both the subject and the caregiver bear the burden of the illness, and the level of their overall distress may be high. Therefore, study procedures pertaining to the tDCS procedure and data collection have to be user friendly, easy and not time-demanding. Further, time planning of study procedures should leave reasonable margins acceptable for both the dyad of subject-caregiver and the study personnel, for example when scheduling the real-time video monitoring of the procedure. Further, it needs to be taken to the account that the tDCS stimulation usually takes 15–20 min during which the subject has to remain seated or in the bed, without walking around. Therefore, subjects who for variety of reasons, such as restlessness, are not comfortably able to comply with that requirement are not good candidates for the tDCS procedure. Another aspect to consider in this category is data collection, especially in study population involving palliative care subjects at advanced stage of the illness. While data collection in healthy populations may include extensive questionnaire sets and testing, data collection in palliative care subjects should very carefully reflect the specifics of the involved population.
-* Polypharmacy*: Symptom management in palliative care frequently relies on pharmacological treatments, often including multiple medications. There is growing evidence indicating that certain agents (such as NMDA antagonists or amphetamine) may alter (inhibit or enhance) tDCS effects. This requires careful consideration when planning the tDCS protocol, because it is unlikely that medication wash-out prior the study participation would be feasible in palliative care patients.
-* Feasibility*: Overall, the feasibility of the home-delivered remotely-monitored tDCS in palliative care patient population is multifaceted, including (but not limited to) the following elements:
- The patient’s and family caregiver’s understanding of the procedure, their willingness and ability to participate in the study.
- Caregiver’s ability to perform tDCS specific procedures [after training]: Establishing videoconference connection; Assembling the electrodes and the head set; Positioning the headset on the subject’s head; Turning on-off the tDCS unit; and the procedure has to be regarded by the involved caregiver as acceptable for him/her and the subject;
- Subject’s acceptability and tolerability of the procedure: Able to remain seated or in bed for the 20-min stimulation [does not interrupt the stimulation session by walking around]; Able to provide a brief feedback or numerical rating when asked; Regards the procedure as acceptable; Tolerates the tDCS procedure (in the means of adverse events);
- Home environment: the Internet connectivity sufficient for the videoconference connection; sufficient space to accommodate the tDCS and videoconferencing devices.

## Conclusion

Remotely-supervised tDCS can serve as an extension of in-clinic administered tDCS sessions. Proper frameworks around clinical staff training, user capability, training and monitoring guides are intended to maintain the same level of safety and tolerability experienced within the clinic setting. Careful consideration of each of these criteria is essential to a remotely-supervised trial. Future expansion toward a more robust training method for clinical staff on the technique will further optimize its application. In addition, following the completion of each clinical trial, population-specific modifications will be considered within the training, design, and monitoring of such patient populations. For some subjects or populations, prescription for direct home use may ultimately be the resulting clinical application, while others may be eligible only for continued remotely-supervised use. Validation of the compliance experienced in remotely-supervised tDCS trials will serve as a means of comparison to the level of compliance observed within in-clinic trials. Expanding the patient populations able to comply with a tDCS trial through remote supervision can ultimately broaden the reach of clinical trials, helping to deepen the current understanding of tDCS. With these principles in place, tDCS clinical trials will expand with results to ultimately guide appropriate and effective clinical use.

## Conflict of Interest Statement

CUNY has patents with Marom Bikson as inventor. Marom Bikson has equity in Soterix Medical.
